# Progress in Surgery

**Published:** 1898-08-20

**Authors:** 


### PROGRESS IN SURGERY.

### HERNIA.

(*Continued from page* 340.)

**Tuberculosis of the Hernial Sac.**—There is an interesting editorial note on this condition in the *New York Medical Journal* of March, 1898, with an account of a recently-published case of Dr. Julius Sternberg's. From this it appears that the condition has been several times met with in performing operations for the radical cure of hernia. There are some 35 cases recorded, in three of which the diagnosis was made before operation. It occurs most often in children under 10, and most often there is general tubercular peritonitis, and the type of the disease in the sac is the same as that of the peritoneum, being ascitic, dry, or sacculated. Most commonly there is only a little serum in the sac, but there may be intestine, either matted with thick pus between the coils, or free. Sternberg's case was in a woman of 27. She had had tuberculous pleurisy when 16, and had never quite recovered, and dulness remained. She developed double inguinal hernia seven years later. A truss could not be worn, because of the pain it induced. The larger sac was much thickened with tubercular tissue, and contained serum; the smaller contained only a few tubercles. After radical cure the suture wounds on both sides became tuberculous. At the end of 18 months the patient was in perfect health.

**The Radical Cure of Hernia.**—1. Inguinal: Mr. Stanmore Bishop[5] points out the importance of not attempting to cure chronic hernia while the cause which produced it is still present. He divides hernias into two groups. In one the course is acute, and the circulation in the extruded part is interfered with. In the other the course is chronic, and it is only fæcal circulation which may be effected. These chronic cases cannot be permanently cured by any operation, unless the cause of their production is removed. The writer deprecates the prevailing custom of attributing the failure of radical operations to permanently cure hernias to faulty methods of operation, and the frequency with which new operations are attempted, since the reason of failure is so often not a faulty operation, but the persistence of the original cause of the hernia. It is foolish to expect a permanent cure if there is thoracic or abdominal disease causing chronic cough, or stricture of the rectum or urethra leading to straining with defæcation and urination. Certain operations have similar effects upon the results. In obtaining the histories of patients with chronic hernia, the frequency with which they believe the cause to have been a sudden strain, and do not recognise the more persistent causes at work, is very remarkable, and is well illustrated by Mr. Bishop.

Dr. Archibald Watson[6] lays stress on the restoration of the posterior wall of the inguinal canal after the plan of Bassini. He points out that to pass the deep sutures as Bassini does, requires a needle curved like a fish-hook, and necessitates leaving the knots inside the canal in contact with the spermatic cord. Dr. Hamilton is mentioned as having very successfully, on an occasion, used an ordinary needle and passed the sutures just under Poupart's ligament, then through the conjoined tendon, and out just above the ligament, thus avoiding leaving hard silk-woven gut knots in the canal. The knots in this method are tied under the skin along the base of Scarpa's triangle. Scrotal incisions are deprecated for hernia, varicocele, or castration. Dr. J. Ewart[7] writes favourably of Bennet's modification of Barker's operation. The operation only differs from Barker's in the method of fixing the stump of the sac high up at the internal ring. A Liston's needle is passed through all the layers of the abdominal wall a little above and to the inner side of the internal ring, and out through the divided neck of the sac transfixing its side and threaded and withdrawn. The other end of the silk which is used is again threaded on the needle and passed through the same structures on the outer side in reverse order. Thus a loop of silk lies over the end of the stump. The latter is ligatured and pushed up to the ring with the finger and the silk ligature tied over a small gauze pad lying on the skin to prevent cutting. This loop ligature is removed in a fortnight. The writer does not favour the use of silk or silk gut for ligature of the sac or buried sutures. They frequently give rise to trouble, it may be months afterwards, and have to be removed. The writer lays much stress on the after treatment, by which no strain is thrown upon the wound. This includes a month or six weeks' rest in bed, with hips flexed, early relief of any tympanites by the rectal tube, and careful lifting when the bed pan is used. No work should be done for three months. Dr. G. R. Fowler[8] has recently devised and carried out a new operation for the radical cure. The essential feature of the method is the displacement of the spermatic cord backwards into the peritoneal cavity from the internal ring to opposite the external ring, where it is allowed to emerge. The posterior wall of the inguinal canal, including the transversalis fascia and peritoneum, is divided, the deep epigastric vessels being tied. The sac and cord are previously isolated and the former cut off at the level of the abdominal wall. The peritoneum and transversalis fascia are then united in front of the spermatic cord down to a point opposite the external ring, where the cord is brought out. The canal is then closed and the external ring strengthened by the displacement outwards of the pubic attachment of the rectus muscle. Thus both the internal ring and inguinal canal are obliterated. No other method in vogue accomplishes this.

[5] New York Med. Jour., April, 1898. [6] Australasian Med. Gazette, Dec., 1897. [7] Australasian Med. Gazette, May, 1897. [8] Therapeutic Gazette, 1898.

